# Glucose Metabolism Modification Induced by Radioligand Therapy with [^177^Lu]Lu/[^90^Y]Y-DOTATOC in Advanced Neuroendocrine Neoplasms: A Prospective Pilot Study within FENET-2016 Trial

**DOI:** 10.3390/pharmaceutics14102009

**Published:** 2022-09-22

**Authors:** Luca Urso, Stefano Panareo, Angelo Castello, Maria Rosaria Ambrosio, Maria Chiara Zatelli, Matteo Caracciolo, Eugenia Tonini, Giorgia Valpiani, Alessandra Boschi, Licia Uccelli, Corrado Cittanti, Mirco Bartolomei

**Affiliations:** 1 Department of Translational Medicine, University of Ferrara, 44124 Ferrara, Italy; 2 Nuclear Medicine Unit, Oncological Medical and Specialist Department, University Hospital of Ferrara, 44124 Ferrara, Italy; 3 Nuclear Medicine Unit, Oncology and Haematology Department, University Hospital of Modena, 41125 Modena, Italy; 4 Nuclear Medicine Unit, Fondazione IRCCS Ca’ Granda, Ospedale Maggiore Policlinico, 20122 Milan, Italy; 5 Department of Medical Sciences, University of Ferrara, 44124 Ferrara, Italy; 6 Medical Physics Unit, University Hospital of Ferrara, 44124 Ferrara, Italy; 7 Research Innovation Quality and Accreditation Unit, University Hospital of Ferrara, 44124 Ferrara, Italy; 8 Department of Chemical, Pharmaceutical and Agricultural Sciences, University of Ferrara, 44121 Ferrara, Italy

**Keywords:** neuroendocrine neoplasms, NEN, NET, radioligand therapy, RLT, peptide receptor radionuclide therapy, PRRT, 18F-FDG PET/CT, FDG PET, therapy response evaluation

## Abstract

[^18^F]F-FDG (FDG) PET is emerging as a relevant diagnostic and prognostic tool in neuroendocrine neoplasms (NENs), as a simultaneous decrease in [^68^Ga]Ga-DOTA peptides and increase in FDG uptake (the “flip-flop” phenomenon) occurs during the natural history of these tumors. The aim of this study was to evaluate the variations on FDG PET in NEN patients treated with two different schemes of radioligand therapy (RLT) and to correlate them with clinical–pathologic variables. A prospective evaluation of 108 lesions in 56 patients (33 males and 23 females; median age, 64.5 years) affected by NENs of various primary origins (28 pancreatic, 13 gastrointestinal, 9 bronchial, 6 unknown primary (CUP-NENs) and 1 pheochromocytoma) and grades (median Ki-67 = 9%) was performed. The patients were treated with RLT within the phase II clinical trial FENET-2016 (CTID: NCT04790708). RLT was offered for 32 patients with the MONO scheme (five cycles of [^177^Lu]Lu-DOTATOC) and for 24 with the DUO scheme (three cycles of [^177^Lu]Lu-DOTATOC alternated with two cycles of [^90^Y]Y-DOTATOC). Variations in terms of the ΔSUVmax of a maximum of three target lesions per patient (58 for MONO and 50 for DUO RLT) were assessed between baseline and 3 months post-RLT FDG PET. In patients with negative baseline FDG PET, the three most relevant lesions on [^68^Ga]Ga-DOTA-peptide PET were assessed and matched on post-RLT FDG PET, to check for any possible changes in FDG avidity. Thirty-five patients (62.5%) had at least one pathological FDG uptake at the baseline scans, but the number was reduced to 29 (52%) after RLT. In the patients treated with DUO-scheme RLT, 20 out of 50 lesions were FDG positive before therapy, whereas only 14 were confirmed after RLT (*p* = 0.03). Moreover, none of the 30 FDG-negative lesions showed an increased FDG uptake after RLT. The lesions of patients with pancreatic and CUP-NENs treated with the DUO scheme demonstrated a significant reduction in ΔSUVmax in comparison to those treated with MONO RLT (*p* = 0.03 and *p* = 0.04, respectively). Moreover, we found a mild positive correlation between the grading and ΔSUVmax in patients treated with the MONO scheme (r = 0.39, *p* < 0.02), while no evidence was detected for patients treated with the DUO scheme. Our results suggest that RLT, mostly with the DUO scheme, could be effective in changing NEN lesions’ glycometabolism, in particular, in patients affected by pancreatic and CUP-NENs, regardless of their Ki-67 index. Probably, associating [^90^Y]Y-labelled peptides, which have high energy emission and a crossfire effect, and [^177^Lu]Lu ones, characterized by a longer half-life and a safer profile for organs at risk, might represent a valid option in FDG-positive NENs addressed to RLT. Further studies are needed to validate our preliminary findings. In our opinion, FDG PET/CT should represent a potent tool for fully assessing a patient’s disease characteristics, both before and after RLT.

## 1. Introduction

Neuroendocrine neoplasms (NENs) are a heterogeneous group of tumors, arising from diffuse neuroendocrine system cells [1]. These neoplasms can potentially occur from any part of the body, even though the entero-pancreatic district is the most common site of disease, including about two thirds of all NENs [2,3]. NENs can have a wide-ranging spectrum of clinical behavior, presenting as indolent well-differentiated tumors or aggressive and poorly differentiated cancers [4].

Beyond the primary role of histological and immuno-histochemical tests for a proper biological classification of the neoplasm, imaging-based techniques may play a pivotal role to help clinicians both in choosing the best patient-tailored therapeutic option and in managing the response to treatment. Well-differentiated NENs usually overexpress somatostatin receptors (SSTr) on their cells, providing a suitable target for radiolabeled somatostatin analogues (SSTa) [5]. Therefore, among imaging techniques, nuclear medicine functional investigations play a main role. According to a theranostic approach, SSTa can bind a positron-emitting isotope, [^68^Ga]Ga, for diagnostic purposes ([^68^Ga]Ga-DOTA-SSTa PET/CT) or a high-energy beta emitter, such as [^90^Y]Y or [^177^Lu]Lu, for radioligand therapy (RLT) [6]. In particular, [^68^Ga]Ga-DOTA-SSTa PET/CT contributes to the initial characterization of the neuroendocrine lesions, to the staging and re-staging of the NEN patients over time and, above all, to the selection of those patients who will be candidates for RLT [7,8].

The role of [^18^F]F-FDG (FDG) PET/CT in NENs has gained a progressive relevance in recent years. Several studies have demonstrated a correlation between increasing FDG avidity and dedifferentiation changes in NEN cells, making FDG PET/CT a potential baseline prognostic test [2,4,9]. The reason behind the progressive increase in FDG avidity in advanced NENs has to be related to the heterogeneity of tumor cells. In a certain tumor mass, some aggressive FDG avid clones—characterized by a predominant anaerobic glycolytic activity (known as the “Warburg effect”)—may lay beside more indolent clones, marked by a high cell expression of SSTr [10,11,12,13]. As the disease progresses, a “flip-flop” phenomenon has been described, with a progressive decrease in SSTr expression on [^68^Ga]Ga-DOTA-SSTa PET/CT and a parallel increase in GLUT-1 density and consequently in FDG avidity [4,14,15,16].

The proliferation index, expressed by Ki-67, just indicates a punctual representation of the disease in a certain site of the lesion at a specific time point, but it does not necessarily reflect the current situation for the whole tumor burden [17]. With this in mind, FDG PET/CT might be considered a complementary tool to be associated with [^68^Ga]Ga-DOTA-SSTa PET/CT to have an all-round evaluation of the disease burden.

As for the assessment of the response to RLT, response-evaluation criteria in solid tumors (RECIST) 1.1, applied to contrast enhanced computed tomography (ceCT), is indicated as the main tool by current ENETS guidelines [18]. However, several data in the literature have reported questionable reliability for the morphologic parameters. In particular, the pseudo-progression phenomenon, related to temporary radiation-induced inflammation or necrosis, rather than due to effective disease progression, has been described as a source of error for response assessment [4,19]. Another issue is the delayed morphological response in comparison with the molecular/functional one [5,20]. Therefore, new response-evaluation tools are needed, in particular, when assessing the response to NEN therapy, including RLT [21].

Based on the above-mentioned premises, the aim of our study was to evaluate whether RLT could induce modifications in advanced NEN lesions’ glycometabolism on FDG PET/CT.

The secondary aims were to assess the possible concordance of the RLT response evaluated with [^68^Ga]Ga-DOTA-SSTa and FDG PET/CT, and, finally, whether two different therapeutic schemes, offered within our clinical trial (FENET 2016), might provide different FDG responses to treatment in NENs according to a) primary origin and b) clinical and pathological variables.

## 2. Materials and Methods

This is a pilot study within the prospective phase II clinical trial FENET-2016 (EudraCT: 2016-005129-35—Clinical Trials ID: NCT04790708), currently ongoing at the University Hospital of Ferrara, Italy. The current study has been conducted following the approval of the local institutional ethical committee (“Comitato Etico Unico della Provincia di Ferrara”, Protocol N° 160990 approved on the 13 October 2016) and in accordance with the Declaration of Helsinki and Good Clinical Practice guidelines. Written informed consent was obtained from every patient.

### 2.1. Patients Identification

At the time of the study, 140 patients were enrolled in the FENET-2016 trial on the basis of the following inclusion criteria: (a) established diagnosis of advanced NEN according to the ENETS criteria [22]; (b) positive [^68^Ga]Ga-DOTA-SSTa PET/CT performed within 2 months from the RLT’s first cycle; (c) a radiological examination (ceCT/MRI) and an FDG PET/CT scan performed within 2 months before starting RLT; (d) age ≥ 18. Further details regarding the FENET-2016 trial are available on the trials page on the clinicaltrials.gov website [23].

At the time of the present study, 90 patients had already completed RLT. Among those, 60 pts were suitable for this preliminary pilot study since they had already performed their first follow-up within 3 months after the end of RLT. The follow-up consisted of clinical and instrumental evaluation, with the latter including ceCT/MRI, [^68^Ga]Ga-DOTA-SSTa and FDG PET/CT. Four patients were excluded due to their primary brain disease, poorly evaluable with FDG PET/CT. The remaining 56 patients were thus considered.

### 2.2. Therapy Protocol

A minimum of 30 days of washout from any previous therapy was required, except for cold somatostatin analogues, which were withdrawn only in the 14 days preceding the therapeutic infusion of radiopharmaceuticals. Prior to every therapy administration, a blood routine was performed, to evaluate the patient’s eligibility for therapy and exclude hematological and renal impairment.

RLT was proposed with two mutually exclusive therapeutic schemes, tailored empirically to each patient’s characteristics and extension of disease. The first, the “MONO” scheme, comprised 5 cycles of 3.7–5.55 GBq of [^177^Lu]Lu-DOTATOC, with a cumulative activity of 18.5–27.75 GBq (500–750 mCi); the second, the “DUO” scheme, comprised 3 cycles of 3.7–5.55 GBq of [^177^Lu]Lu-DOTATOC alternated with 2 cycles of 1.85–2.75 GBq of [^90^Y]Y-DOTATOC, for a cumulative activity of 11.1–16.65 GBq of [^177^Lu]Lu-DOTATOC and 3.7–5.55 GBq of [^90^Y]Y-DOTATOC. Between every cycle, an interval of 8–10 weeks was observed.

The main criteria guiding the choice of the therapy scheme were (a) the lesion size; (b) the grading; and (c) comorbidities (such as carcinoid syndrome; impaired renal, hepatic or cardiac functionality; diabetes; and hypertension). In particular, the “MONO” scheme was preferred in the presence of prevalent small lesions, low grading and relevant concomitant disease/affections. On the contrary, the “DUO” scheme was proposed in patients presenting large and aggressive lesions. Despite these premises, the choice of the treatment scheme was—albeit marginally—conditioned by the commercial availability of the 2 radioisotopes used: [^90^Y]Y and [^177^Lu]Lu. The treatment selection, balanced between the patient clinical status and tumor intrinsic biology, was discussed and shared by the institutional NEN multidisciplinary board, in which nuclear medicine physicians, endocrinologists, surgeons, oncologists, gastroenterologists, radiotherapists and radiologists are all represented.

### 2.3. Response Assessment and Follow-Up

The response assessment was performed 3 months after the last cycle of RLT and included a clinical evaluation of the patient, the repetition of the ceCT/MRI scan, [^68^Ga]Ga-DOTA-SSTa and FDG PET/CT, which had to be compared with the baseline studies.

### 2.4. Image Acquisition

The patients were required to fast for at least 6–8 h and maintain adequate hydration before the FDG PET/CT scans. Diabetic patients had their blood glucose measured before FDG delivery, and those with values above 200 mg/dL were rescheduled. Images were acquired 50–70 min after FDG injection (3.5 MBq/Kg) using a standard technique on a dedicated 3D PET/CT system (Biograph mCT Flow; Siemens Medical Solutions, Malvern, PA, USA). A concomitant low-dose CT scan (120 kV and 80 mA/s) was performed for the attenuation correction of the PET emission data acquired from the mid-thigh to the skull vertex.

[^68^Ga]Ga-DOTATOC PET/CT was performed 50–70 min after the intravenous administration of a mean dose of 150 ± 50 MBq of [^68^Ga]Ga-DOTATOC, using the same tomograph and acquisition protocol described above. The two PET/CT scans were obtained on two different days, within three weeks.

### 2.5. Image Review

The PET/CT images were all processed and analyzed by using a Syngo.via Workstation (Siemens Healthineers, Enlargen, Germany). The PET/CT images were all assessed by two experienced board-certified nuclear medicine physicians. The criteria for a positive finding in the PET/CT studies were (a) focal area(s) of increased tracer uptake or diffusely increased uptake, excluding sites of physiological distribution, in comparison with surrounding tissues. Patients were considered “FDG positive” if at least a positive finding was detected on FDG PET/CT.

A maximum of 3 target lesions were selected for every patient on baseline FDG PET/CT scan, including a primary lesion (Target 1), lymph node metastasis (Target 2) and metastasis at distance (Target 3).

In patients with negative FDG PET/CT at baseline, the ROI corresponding to the most relevant lesion on [^68^Ga]Ga-DOTA-SSTa PET/CT was shifted on follow-up FDG PET/CT imaging series, in order to evaluate any possible change in FDG uptake on the target lesions.

**Figure 1:**
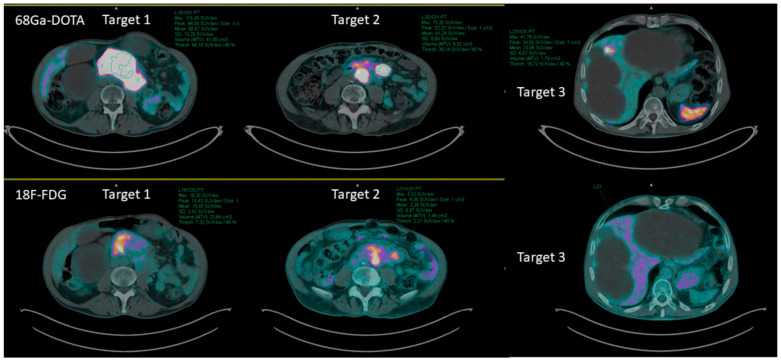
The figure shows the selection process for the target lesions on baseline FDG and [^68^Ga]Ga-DOTATOC PET/CT in a patient of the series. Target 1 was selected on the pancreatic primary lesion, and target 2, on a loco-regional lymph node metastasis. In this case, target 3 was identified only on [^68^Ga]Ga-DOTATOC PET/CT, as the liver metastasis showed no FDG avidity. However, the same target was re-evaluated on follow-up FDG PET/CT to identify any possible glycometabolic variation.

In the case of multiple lesions for each district, the most representative one—in terms of the extension and tracer uptake intensity—was selected (Figure 1).

**Figure 2:**
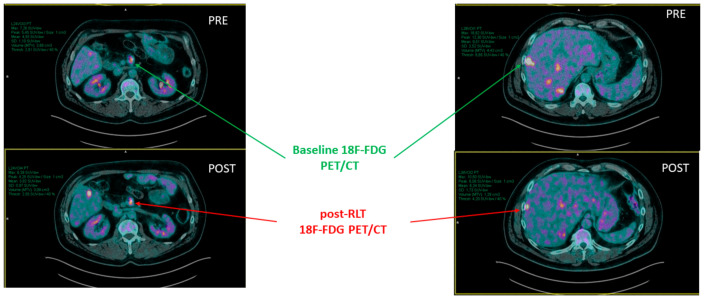
The figure shows the direct comparison made between baseline and post-RLT FDG PET/CT scans, in terms of SUVmax variation (ΔSUVmax) for target 2 and target 3. In this patient, target 1 was not evaluated since the patient had received surgery on the primary lesion.

For each lesion, the maximum standardized uptake value (SUVmax) was calculated and compared between baseline and post-RLT FDG PET/CT scans (ΔSUVmax) (Figure 2). Similarly, the SUVmax and ΔSUVmax of the same target lesions were calculated on every [^68^Ga]Ga-DOTATOC PET/CT.

### 2.6. Statistical Analysis

The normality of the distribution of the continuous variables was assessed with the Shapiro–Wilk test. In the case of symmetric distributions, the variables are represented with the mean and standard deviation (SD), while for non-normal distributions, the median value and interquartile range [1Q 3Q] are used; categorical data are expressed as total numbers and percentages.

Mcnemar’s test for the significance of changes was performed separately for the “MONO” and “DUO” treatments to assess whether lesions became negative or became positive following therapy.

The Kruskal–Wallis test was used to evaluate the presence of differences in lesions’ ΔSUVmax on FDG PET/CT after RLT, divided by origin and for every target. The Mann–Whitney test was used to evaluate the presence of differences, divided by origin, between the variations of the lesions treated with the “MONO” or “DUO” schemes. Using Spearman’s rank correlation coefficient (ρ), the correlation between Ki-67, age and [^90^Y]Y activity was calculated with the variation of FDG PET/CT, divided by the “MONO” and “DUO” therapy schemes.

Wilcoxon’s paired test was used to evaluate the differences in median lesion changes between FDG PET/CT and [^68^Ga]Ga-DOTATOC PET/CT, divided by target, for both therapy schedules.

All the analyses were performed using Stata 15.1 SE (Stata Corporation, College Station, TX, USA). A *p* value < 0.05 was defined as statistically significant.

## 3. Results

Overall, 56 patients (33 males and 23 females; median age, 64.5 years) affected by advanced NENs of various origins (28 pancreas, 13 gastrointestinal, 9 lung, 6 unknown primary (CUP-NENs) and 1 pheochromocytoma) and grades (9 G1, 43 G2, 3 G3 and 1 unknown) were enrolled. The detailed patients’ characteristics are described in Table 1. Statistical elaboration of the data confirmed the homogeneity of the population.

Among the 56 patients selected, 32 were treated with the MONO scheme and received a median cumulative activity of 24.4 GBq (range, 23.47–25.75 GBq). The remaining 24 patients received RLT with the DUO scheme, for a median cumulative activity of 19.7 GBq (range, 19.18–20.7 GBq), distributed in 14.24 GBq of [^177^Lu]Lu (range, 13.8–15.19 GBq) and 5.47 GBq of [^90^Y]Y (range, 5.2–5.96 GBq).

**Figure 3:**
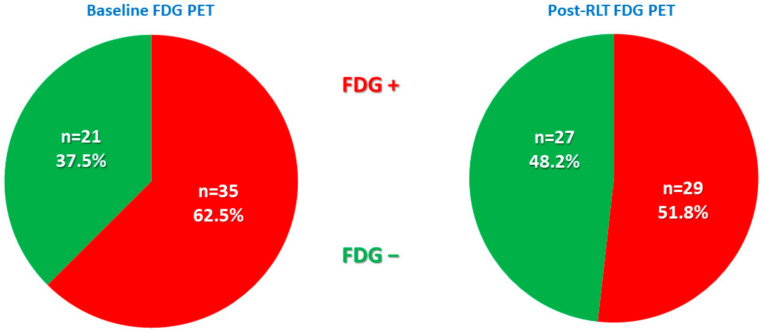
Graphic representation of patients’ positive vs. negative FDG PET/CT at baseline and post-RLT scan.

A total of 35 patients (62.5%) had an FDG-positive scan before RLT, while 21 were FDG negative (37.5%). After RLT, 6 more patients (*n* = 27; 48.2%) demonstrated a complete negative FDG PET/CT scan, while 29 (51.8%) remained positive (Figure 3).

A total of 108 lesions were evaluated. The numbers of lesions studied in patients treated with the MONO and DUO schemes were 58 (53.7%) and 50 (46.3%), respectively (Table 2).

In the patients included in the MONO scheme treatment, 31 lesions (54.4%) were FDG positive and 27 (45.6%) FDG negative at baseline. After RLT, 4 lesions shifted from FDG positive to negative and 3 lesions from FDG negative to positive, with a total of 30 lesions turning out to be FDG positive and 28 negative. Applying Mcnemar’s test, no statistically significant difference was found. Moreover, among the 27 lesions that remained stably FDG positive despite the treatment, 13 showed an increase in the ΔSUVmax value after therapy, while the remaining 14 had a partial glycometabolic metabolic response.

As for the DUO scheme, 20 lesions were FDG positive (40%) and 30 negative (60%) at the baseline scan. Within the 20 FDG-positive lesions, 6 (30%) showed a negative FDG scan after RLT, while none of the FDG-negative lesions showed a significant FDG increase after treatment (*p* = 0.025). Moreover, 9 out of the 14 lesions that remained FDG positive after RLT had a decrease in ΔSUVmax value, while only 5 showed an increase. The distribution of the results is displayed in Table 2.

Taking into account the FDG variation of the whole number of lesions studied, no statistically significant difference was found according to the NENs’ primary origin. However, when separately considering patients bearing FDG-positive lesions from a pancreatic primary origin, a statistically significant reduction in the median ΔSUVmax on FDG PET/CT was found between those treated with the DUO scheme vs. MONO scheme (−0.41 vs. 0.02, respectively, *p* = 0.036) (Table 3). A similar result was obtained when considering the subset of patients affected by FDG-positive lesions from CUP-NENs treated with the DUO scheme vs. MONO scheme (−1 vs. 0.26, respectively, *p* = 0.044). No significant difference for lesions of NENs from midgut and bronchial primary origins between the two treatment schemes was found.

Analysis was not possible for the single patient with metastatic pheochromocytoma included in the study. However, the patient was treated with the MONO scheme and showed a relevant ΔSUVmax decrease on both PET/CT (−36.5% on the two targets on FDG and −21% on [^68^Ga]Ga-DOTATOC PET/CT) after RLT.

Comparing the ΔSUVmax trends on both FDG PET/CT and [^68^Ga]Ga-DOTATOC, no significant variations between the two different therapy schemes were found for any target. In particular, for the MONO scheme, the median ΔSUVmax was −0.19 for FDG PET/CT and −0.03 for [^68^Ga]Ga-DOTATOC, while for the DUO scheme, the median ΔSUVmax was −1.60 for FDG PET/CT and −0.76 for [^68^Ga]Ga-DOTATOC.

In patients treated with the MONO scheme, a moderate positive correlation (ρ = 0.392, *p* = 0.024) was found between the ΔSUVmax on FDG PET/CT and the mitotic index—expressed by Ki-67—suggesting that more aggressive neoplasms showed a tendency to progression on FDG PET/CT.

Conversely, among the patients treated with the DUO scheme, no significant correlation between Ki-67 and the lesion’s ΔSUVmax on FDG PET/CT was identified (ρ = 0.076, *p* = 0.748).

No significant correlation between age and the lesions’ ΔSUVmax on FDG PET/CT was detected for both treatment schemes.

## 4. Discussion

In recent years, RLT has become widespread as a therapeutic option for advanced, metastatic or inoperable NENs, since the NETTER-1 trial demonstrated a clear survival advantage compared to “cold” SSTa therapy in midgut low-grade NENs [24]. While a positive [^68^Ga]Ga-DOTA-SSTa PET/CT (or eventually 111In-Octreoscan) is mandatory for starting RLT, the role of FDG PET/CT is still debated, and it is currently not recommended by the ENETS guidelines, except in the case of G3 NENs [25,26]. Conversely, several studies recently investigated the potential role of FDG PET/CT in NEN staging, focusing mainly on the prognostic impact of FDG-positive lesions [27,28]. In particular, Binderup et al. [13] recently reported a positive FDG PET/CT as the only identifier of high risk for death in a large cohort of gastro-entero-pancreatic (GEP) NENs, proposing differentiating G1 and G2 tumors into low- and high-risk groups depending on FDG-positive or negative scans. Moreover, the same authors also reported a prolonged OS and PFS in FDG-positive patients receiving RLT compared to patients receiving other kinds of therapies (4.4 vs. 1.4 years, *p* = 0.001). Nevertheless, only a few other studies systematically assessed RLT-induced changes on follow-up FDG PET/CT [1,29]. Oh et al. [1] reported for the first time that RLT could also have a therapeutic effect on the glucose metabolism of NEN lesions, as long as they showed a synchronous SSTr expression on [^68^Ga]Ga-DOTA-SSTa PET/CT. Moreover, Nilica et al. [29] reported that a stable or decreased lesion SUVmax on FDG PET/CT after RLT was associated with a good prognosis. Conversely, in three patients who died shortly after RLT, the SUVmax value was increased by at least ≥40% from the baseline values.

The present study is consistent with data reported above confirming that RLT can induce relevant metabolic variations on FDG-positive lesions in patients affected by NENs of various primary origins. Our most impacting result was that six patients evolved from FDG positive to FDG negative after RLT, in what could be considered a positive response to treatment in terms of outcome.

In the past, the “law of Bergonié and Tribondeau” described that highly proliferating cells are more sensitive to radiation damage [30]. This statement was confirmed in more recent studies assessing external beam radiation therapy (EBRT) outcomes in different cancers. For instance, Ishibashi et al. [31] reported that a high Ki-67 proliferation index was a predictive factor for a complete response after radiation therapy in small cell lung cancer.

Commonly, NEN lesions are widely heterogeneous in terms of cell population, since clusters of well-differentiated elements overexpressing SSTr frequently locate beside other undifferentiated FDG-positive clones, which are supposed to have a lower or absent SSTr expression and a higher proliferation index [12]. Despite radiopeptides being selectively accumulated by those cells overexpressing SSTr, they can also damage nearby undifferentiated cells with less SSTr expression by the crossfire effect. In this context, [^90^Y]Y-DOTATOC β-particles could be more effective than [^177^Lu]Lu-DOTATOC ones, thanks to their higher emission energy and tissue penetration, which enhances the crossfire effect [32,33,34,35]. As a result, we can speculate that DUO-scheme RLT might be able to clear-cut NEN lesions from FDG-positive cell clones. Indeed, in our study, patients treated with the DUO scheme showed a significant therapeutic benefit regarding FDG-positive lesions, since 30% of these showed a complete FDG negativization (*p* = 0.025), and 9 out of the 14 who remained FDG positive after RLT had a decreased ΔSUVmax.

Despite its pioneering role, [^90^Y]Y-based RLT has been employed discontinuously, mainly because of its proven correlation with renal toxicity [36] and because of the concomitant introduction of [^177^Lu]Lu radiopeptides [37]. In the last 20 years, [^177^Lu]Lu-DOTATATE/TOC has mainly been employed for RLT in NENs and demonstrated efficacy and safety [6,33]. However, some authors have restored [^90^Y]Y-based RLT in mixed therapeutic schemes with [^177^Lu]Lu radiopeptides [38,39,40,41,42]. The combined use of the two radionuclides could theoretically ensure a better risk/benefit ratio in FDG-positive patients, being effective on a wider range of lesions while preserving a safe dosimetric profile.

Our experience suggests that the “DUO” therapy scheme could potentially be preferred particularly in intermediate aggressive NENs, which present some FDG-positive lesions. Nevertheless, a full FDG negativization of 4 lesions and a ΔSUVmax reduction of 14 lesions were found in patients treated with the “MONO” scheme. Although no statistically significant correlation was found, a metabolic response obtained in some lesions treated with the “MONO” scheme suggests that this therapy scheme may also induce changes in FDG-positive lesions. Therefore, we can speculate that [^177^Lu]Lu single-agent RLT probably represents a valid therapeutic approach in patients with mild and restricted FDG tumor burdens and with concomitant comorbidities (renal impairment, hypertension and diabetes). However, further studies with a larger number of lesions investigated are required to confirm this hypothesis.

Several studies investigated the relationships between NEN primary origin and RLT response. Particularly, pancreatic NENs seem to have better outcomes after RLT compared to other GEP-NENs, while CUP-NENs probably have an intermediate response rate [43]. Moreover, neoadjuvant RLT seems promising in allowing second-step surgery in inoperable or borderline-operable pancreatic NENs [44,45]. On the other hand, bronchial origin is reported to be related to a poorer prognosis in comparison to other origins, even though RLT seems to provide a favorable outcome when compared to other systemic therapies that can be offered in this subset of patients [46,47,48]. When considering the entire cohort of patients of this study, no statistically significant differences in the response to RLT were identified by FDG PET/CT between different NEN primary origins. However, we found that pancreatic NENs had a better response, in terms of FDG ΔSUVmax reduction, if treated with the “DUO” scheme (*p* = 0.036). Interestingly, CUP-NENs also showed a similar glycometabolic outcome after RLT (*p* = 0.044), even though this subgroup analysis was affected by the low number of lesions studied. Probably, this finding has to be correlated with the higher energy charge of [^90^Y]Y-radiopeptides delivered to lesions, even though the exact underlying pathophysiological mechanism deserves to be further investigated. The message emerging from these considerations might suggest considering the use of combined RLTs in FDG-positive pancreatic and, probably, CUP-NENs.

Grading, expressed by Ki-67, is firmly considered a strong prognostic factor in NENs [9,49]. The literature reports an inverse correlation between Ki-67 and therapeutic outcomes in terms of overall survival and the disease control rate [38,49,50,51]. For these reasons, we expected to find a positive correlation between Ki-67 and ΔSUVmax increase on FDG PET/CT in our study. As a result, a moderate positive correlation was found (ρ = 0.392, *p* = 0.024) only in patients treated with the MONO scheme. Thus, we hypothesize that patients with higher Ki-67 could present an increased risk of metabolic progression on FDG PET/CT when treated with [^177^Lu]Lu-based RLT alone. Interestingly, no correlation was found between Ki-67 and ΔSUVmax on FDG PET/CT in patients treated with the DUO-therapy scheme. These data prompt us to speculate that this outcome might be related to a better effectiveness of combined RLT in controlling FDG-positive lesions, which are more frequent in patients with a higher Ki-67 index. Of course, larger cohort studies with longer follow-up observations are needed to prove our assumption.

Furthermore, we did not find significant correlation between age and ΔSUVmax on FDG PET/CT for both treatment schemes. This seems to suggest that age may not be a relevant prognostic factor when assessing lesion glycometabolism in patients treated with RLT, as long as the patient’s performance status is good enough to carry out the complete course of therapy.

In 2011, Oh et al. [1] firstly showed a significant correlation between ΔSUVmax on FDG and that on [^68^Ga]Ga-DOTATOC PET/CT in response to RLT. In our study, we could not confirm this type of correlation. More frequently, a discrepancy—in the same patient—between the ΔSUVmax on the two different PET examinations was seen in this study. In our opinion, the most interesting scenario, for its potential prognostic impact, was that presenting a negativization on FDG PET/CT even in the presence of a persistent positive [^68^Ga]Ga-DOTATOC PET/CT. This result could be considered as a global downgrading of disease determined by the elimination of high-grade clones and the mutual selection of well-differentiated cells, which are probably less sensitive to the irradiation insult. Hence, keeping in mind the limitations of the RECIST-based response assessment, FDG PET/CT should be considered a useful tool for obtaining a complete evaluation of the response to RLT, at least until more specific biomarkers (such as the NETest) become widely available for staging and restaging [52,53]. Therefore, our suggestion is to perform a dual-tracer PET evaluation at least during the early follow-up after RLT.

Our study presents a few limitations. Firstly, we decided to evaluate RLT-induced modifications only on functional imaging, excluding morphological evaluation using RECIST criteria on CT. This choice was taken to avoid possible inconsistencies between morphological and functional imaging, due to the short time of follow-up (3 months) and the relatively high possibility of radiation-induced pseudo-progression on CT [4,5,19,54]. Of note, we did not consider patient outcomes because the aim of this study was to assess glycometabolic changes in NEN lesions following RLT and eventually to report any differences between two alternative therapy schemes. Another limitation is that our analysis was performed using only the SUVmax parameter and in a relative small cohort of patients. Further studies with a larger sample size and considering the newest volumetric parameters (such as the total lesion glycolysis—TLG—and metabolic tumor volume—MTV), which have recently shown encouraging preliminary results, could add more relevant information about this issue [55,56,57].

## 5. Conclusions

Our study suggests a possible role for RLT in inducing modifications in NENs presenting FDG avid lesions. In particular, we found that combined RLT, offered with alternated cycles of [^177^Lu]Lu- and [^90^Y]Y-labelled radiopeptides, seems to be more effective on FDG-positive NEN lesions in comparison to [^177^Lu]Lu-RLT alone. Furthermore, our results highlight that pancreatic and, apparently, CUP-NENs seem to have better outcomes if treated with combined RLT, in terms of FDG lesion metabolism reduction. Moreover, the DUO RLT response, evaluated with FDG PET/CT, does not show any correlation with the Ki-67 index, while a moderate positive correlation was found in lesions in patients treated with MONO RLT. This evidence should lead to a preferential choice of combined RLT in patients with higher tumor grades, with pancreatic origins and presenting FDG avid lesions. Further prospective studies with longer follow-up are needed to verify our preliminary evidence. In our experience, FDG PET/CT represents a potent tool for fully assessing a patient’s disease characteristics, both before and after RLT.

## Figures and Tables

**Table 1:** Population of the study.

Patients		Mono (32)	Duo (24)	Total (56)	*p*-Value
Gender, *n* (%)					ns
	Male	18 (56.3)	15 (62.5)	33 (58.9)	
	Female	14 (43.7)	9 (37.5)	23 (41.7)	
Age, median [IQR]		60 [48 69]	68 [58.5 73]	64.5 [52 71]	0.018
Origin	I	7 (21.9)	3 (12.5)	11 (19.3)	ns
	P	14 (43.7)	14 (58.3)	28 (49.1)	ns
	R	1 (3.1)	1 (4.2)	2 (3.5)	ns
	B	5 (15.6)	4 (16.7)	9 (15.8)	ns
	U	4 (12.5)	2 (8.3)	6 (10.5)	ns
	SA	1 (3.1)	0 (0)	1 (1.7)	ns
Grading (%)	G1	6 (19.3)	3 (12.5)	9 (16.4)	ns
	G2	24 (77.4)	19 (79.2)	43 (78.2)	ns
	G3	1 (3.3)	2 (8.3)	3 (5.5)	ns
Functioning	Yes	15 (75)	5 (25%)	20 (35.7)	ns
	No	17 (47.2)	19 (52.8)	36 (64.3)	ns

I: ileum NENs; P: pancreatic NENs; R: rectum NENs; B: bronchial NENs; U: unknown primary origin NENs (CUP-NENs); SA: sympathetic–adrenergic axis NENs; ns: non-significant.

**Table 2:** Overall FDG lesion modification according to therapeutic scheme.

	Baseline		POST-RLT		TOTAL	*p* Value
	FDG+	FDG−	FDG+	FDG−		
MONO	31	27	30	28	58 (56.7%)	0.705
DUO	20	30	14	36	50 (46.3%)	0.025
TOTAL	51	57	44	64	108 (100%)	

**Table 3:** Analysis of stratified median ΔSUVmax on FDG PET/CT for primary origin in MONO vs. DUO RLT. The single patient with metastatic pheochromocytoma is not included.

	ΔSUVmax FDG PET/CT	MONO	DUO	*p* Value
Origin, median [IQR]	Pancreas	0.02 [−0.23 1]	−0.41 [−0.65 −0.09]	0.036
	Bronchial	−0.14 [−0.35 0.5]	−0.4 [−1 0.19]	0.615
	Ileum + rectum	−0.36 [−0.7 0.07]	0.19 [0.17 0.21]	0.354
	Unknown primary origin	0.26 [−0.04 0.67]	−1 [−1 −1]	0.044

## Data Availability

The data presented in this study are available on motivated request to the corresponding author.
